# *Bacillus* Pectinases as Key Biocatalysts for a Circular Bioeconomy: From Green Extraction to Process Optimization and Industrial Scale-Up

**DOI:** 10.3390/biotech14030074

**Published:** 2025-09-19

**Authors:** Fatima Zohra Kaissar, Khelifa Bouacem, Mohammed Lamine Benine, Sondes Mechri, Shubha Rani Sharma, Vishal Kumar Singh, Mahfoud Bakli, Seif El Islam Lebouachera, Giovanni Emiliani

**Affiliations:** 1 Synthesis of Environmental Information Laboratory (LSIE), Djillali Liabes University, Sidi Bel Abbes 22000, Algeria; benineamine@yahoo.fr; 2 Laboratory of Cellular and Molecular Biology (LCMB), Microbiology Team, Faculty of Biological Sciences (FSB), University of Sciences and Technology Houari Boumediene (USTHB), Bab Ezzouar, Algiers 16000, Algeria; khelifa.bouacem@ummto.dz; 3 Department of Biochemistry and Microbiology, Faculty of Biological and Agricultural Sciences (FBAS), University Mouloud Mammeri of Tizi-Ouzou (UMMTO), Tizi-Ouzou 15000, Algeria; 4 Laboratory of Microbial Biotechnology and Engineering Enzymes (LMBEE), Centre of Biotechnology of Sfax (CBS), University of Sfax, Sfax 3018, Tunisia; sondes.mechri@yahoo.com; 5 Department of Bioengineering and Biotechnology, Birla Institute of Technology, Mesra, Ranchi 835215, Jharkhand, India; srsharma@bitmesra.ac.in; 6 Department of Biotechnology, Ranchi University, Ranchi 834001, Jharkhand, India; vsevishal67@gmail.com; 7 Laboratoire de Valorisation et Conservation des Écosystèmes Arides (LVCEA), Faculté des Sciences de la Nature et de la Vie et Sciences de la Terre, Université de Ghardaia, B.P. 455, Ghardaïa 47000, Algeria; bakli.mahfoud@univ-ghardaia.dz; 8 Institut des Sciences Analytiques et de Physico-Chimie Pour l’Environnement et les Matériaux (CNRS, IPREM), Université de Pau et des Pays de l’Adour, 2 Avenue P. Angot, Technopôle Hélioparc, 64000 Pau, France; 9 Institute for Sustainable Plant Protection, National Research Council of Italy (CNR-IPSP), Via Madonna del Piano 10 Sesto Fiorentino, 50019 Firenze, Italy; giovanni.emiliani@cnr.it

**Keywords:** agro-industrial waste valorization, *Bacillus* spp., circular bioeconomy, industrial biocatalysis, pectin, pectinolytic enzymes, solid-state fermentation, statistical enzyme optimization, thermostable enzymes

## Abstract

Pectins are high-value plant cell-wall polysaccharides with extensive applications in the food, pharmaceutical, textile, paper, and environmental sectors. Traditional extraction and processing methodologies rely heavily on harsh acids, high temperatures, and non-renewable solvents, generating substantial environmental and economic costs. This review consolidates recent advances across the entire *Bacillus*–pectinase value chain, from green pectin extraction and upstream substrate characterization, through process and statistical optimization of enzyme production, to industrial biocatalysis applications. We propose a practical roadmap for developing high-efficiency, low-environmental-footprint enzyme systems that support circular bioeconomy objectives. Critical evaluation of optimization strategies, including submerged versus solid-state fermentation, response surface methodology, artificial neural networks, and design of experiments, is supported by comparative data on strain performance, fermentation parameters, and industrial titers. Sector-specific case studies demonstrate the efficacy of *Bacillus* pectinases in fruit-juice clarification, textile bio-scouring, paper bio-bleaching, bio-based detergents, coffee and tea processing, oil extraction, animal feed enhancement, wastewater treatment, and plant-virus purification. Remaining challenges, including enzyme stability in complex matrices, techno-economic scale-up, and structure-guided protein engineering, are identified. Future directions are charted toward CRISPR-driven enzyme design and fully integrated circular-economy bioprocessing platforms.

## 1. Introduction

The global shift toward sustainable industrial processes and away from fossil-based economies has placed biotechnology at the forefront of the circular bioeconomy [1]. Recent policy frameworks increasingly emphasize waste valorization, promoting the conversion of agro-industrial residues into renewable bioproducts through sustainable bioprocesses [2,3]. These initiatives reinforce the relevance of microbial enzyme platforms, including *Bacillus*-derived pectinases, as enabling technologies for circular and zero-waste industrial strategies [2].

Microbial enzymes currently constitute the majority of the industrial enzyme market, with bacteria alone accounting for roughly 35% of worldwide enzyme sales and projections [4]. Among these biocatalysts, pectinases, which depolymerize the complex pectic polysaccharides of plant cell walls, underpin critical processes such as fruit-juice clarification, textile bioscouring, paper biobleaching, bioethanol production, and wastewater treatment [5]. Commercially, pectinases encompass approximately 20–25% of the global enzyme market, projected to grow from USD 18.3 billion in 2022 to USD 26.6 billion by 2030 (compound annual growth rate, CAGR around 11%) [6].

Bacterial pectinases offer distinct advantages over their fungal counterparts, including simplified fermentation, shorter production cycles, and robustness across wide temperature and pH ranges [4,7]. In particular, *Bacillus* spp. have emerged as exceptional pectinase producers, contributing nearly 50% of the global enzyme sales through rapid growth, efficient extracellular secretion, and tolerance to extremes of pH, temperature, solvents, and ionic strength [8,9].

Their innate capacity to valorize low-cost agro-industrial residues, such as fruit pomace and sugar-beet pulp, simultaneously lowers production costs and embeds waste streams within a truly circular framework [10], directly aligning with the goals of contemporary bioeconomy policies [2]. These eco-friendly innovations mirror the use of low-cost adsorbents, reducing reliance on harsh chemicals and minimizing environmental footprints [11]. Furthermore, the Generally Recognized as Safe (GRAS) status and native secretion pathways of *Bacillus* streamline downstream recovery and regulatory approval, offsetting *Escherichia coli*’s recombinant-expression advantages [12].

Concurrently, “green” pectin-extraction technologies, spanning enzyme, ultrasound, and microwave-assisted methods to subcritical-water, pulsed-electric-field, and high-pressure treatments, have largely replaced conventional acid hydrolysis, yielding high-purity pectins with energy savings of up to 50% and a significant reduction in chemical waste [6,13].

Integrating these upstream advances with optimized pectinase-production platforms, submerged versus solid-state fermentation, response surface methodology (RSM), artificial neural networks (ANN), and design of experiments (DoE), yields a seamless, waste-minimizing production cycle [14]. These approaches enable simultaneous, multivariable optimization rather than one-factor-at-a-time tweaks, greatly improving efficiency and predictive accuracy in defining ideal fermentation conditions [15].

Beyond substrate extraction and enzyme production, *Bacillus* pectinases drive environmental sustainability by enabling waste valorization and bioremediation [6]. Their use in enzyme consortia, combining pectinases with xylanases, amylases, lipases, and cellulases, has supplanted toxic chemicals in textile and paper processing, improved ruminant-feed digestibility, and enhanced juice clarification efficiency [16,17,18].

This review consolidates recent advances across the *Bacillus*–pectinase value chain, from green pectin extraction (e.g., ultrasound and microwave-assisted, subcritical-water, and enzyme-assisted methods) through process and statistical optimization of *Bacillus* pectinase production to industrial biocatalytic applications, highlighting strategies that maximize yield and molecular integrity while minimizing waste within circular-bioeconomy frameworks.

## 2. Review Methodology

A structured literature search was performed to identify experimental studies reporting pectinase production, statistical optimization, provided quantitative enzyme activity data (volumetric activity U/mL or specific activity U/mg) or sufficient process parameters (substrate, time, temperature, pH), industrial biocatalysis and properties from *Bacillus* spp. Databases searched included Web of Science, Scopus, and Google Scholar. Searches were conducted up to the date of submission using combinations of the following terms: (“pectinase” OR “pectinolytic” OR “pectin lyase” OR “polygalacturonase”) AND (“*Bacillus*” OR “*Bacillus* spp.”) AND (“fermentation” OR “solid-state” OR “SmF” OR “SSF” OR “production” OR “optimization” OR “RSM” OR “ANN” OR “pH” OR “thermostable”). Additional records were retrieved from reference lists of relevant articles and review papers. Inclusion criteria were: original experimental data on pectinase production, characterization, or statistical optimization; quantitative enzyme activity data (volumetric activity U/mL or specific activity U/mg) or sufficient process parameters (substrate, time, temperature, pH); and full text available in English. Exclusion criteria included review-only articles without primary data, patents lacking primary experimental results, and abstracts without full methods or results. Unit handling and standardization: volumetric activities (U/mL) and specific activities (U/mg) were tabulated in separate tables (Table 2 and Table 3, respectively) to avoid misleading conversions. Conversion between U/mL and U/mg requires the total protein concentration of the fermentation broth (or purified fraction), which was not consistently reported.

## 3. Pectic Substances

Pectic substances are complex structural polysaccharides of plant cell walls that contribute to cell adhesion, porosity and defense. Their structural heterogeneity, comprising linear backbones and highly branched side chains, presents a substantial challenge for enzymatic degradation and thus for industrial valorization of pectin-rich residues [19]. Efficient depolymerization therefore requires complementary enzyme activities (pectin lyases, polygalacturonases, pectin methyl-esterases and auxiliary esterases) acting in concert [4,8]. Microbial producers such as *Bacillus* spp., which secrete robust multienzyme repertoires, are thus of clear industrial relevance for producing practical pectinolytic cocktails. The abundance of pectin in agro-industrial wastes (e.g., citrus peel, apple pomace) further motivates process development that maximizes production of these multicomponent enzyme mixtures for circular-bioeconomy applications [6,7].

### 3.1. Structural Characteristics and Classification

Homogalacturonan (HG), the most abundant pectic domain, consists of α-1,4-linked D-galacturonic acid residues and can be heavily methyl-esterified. Rhamnogalacturonan I (RG-I) contains alternating rhamnose-galacturonic acid units with extensive arabinan and galactan side chains, while RG-II is a minor but structurally complex and conserved domain enriched in rare sugars [19,20,21]. These domains are distributed heterogeneously among botanical sources (for example, citrus peel is HG-rich, whereas apple pomace is relatively enriched in RG-I) [7,22]. Such compositional differences determine enzyme susceptibility and therefore dictate the spectrum of catalytic activities required for efficient industrial hydrolysis. Consequently, process development emphasizes optimized production of enzyme cocktails (rather than single-enzyme maximization) to ensure broad substrate turnover [4,8,23].

### 3.2. Physiological Roles and Industrial Relevance

In plants, pectins modulate tissue firmness and texture and participate in defense and cell-wall remodeling during ripening and pathogen attack [19,21]. These biological processes involve targeted secretion of native pectinases that selectively modify the pectic matrix. Industrial enzyme applications exploit the same biochemical principles: controlled pectin hydrolysis is used to clarify juices, modify pulp, or remove surface pectins in bioscouring [24]. The specific substrate structure (degree of esterification and side-chain composition) thus determines which catalytic activities and process conditions are appropriate. Accordingly, rational selection of enzyme activities and host platforms, guided by the pectin chemistry of the feedstock, is essential for predictable and scalable valorization of pectin-rich wastes.

### 3.3. Sources and Eco-Friendly Extraction

**Figure 1:**
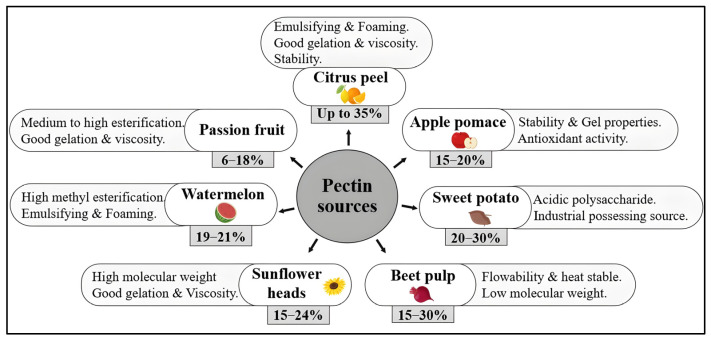
Major plant-based sources of pectin, with approximate pectin content (%) and key functional properties.

Commercial pectins are predominantly recovered from citrus peels and apple pomace, by-products of the juice industry, owing to their high galacturonan content and year-round availability [7]. Emerging sources, including mango peels, sugar beet pulp, sunflower heads, and banana skins, further valorize agro-industrial residues while reducing waste generation (Figure 1) [25]. The chemical profile of pectin from these waste streams varies significantly [7,25]; this variability directly influences the specific cocktail of pectinase activities required for its efficient degradation and thus informs selection or engineering of optimal *Bacillus* production strains [4,23].

#### Green Extraction Techniques

Traditional pectin extraction (mineral-acid hydrolysis, 75–100 °C; alcohol precipitation) is effective but energy-intensive and environmentally burdensome (acidic effluents, solvent usage) (Table 1) [26]. During the past decade, several greener methods have emerged to overcome these limitations:

- Ultrasound-Assisted Extraction (UAE) and Microwave-Assisted Extraction (MAE), these use acoustic cavitation and dielectric heating to disrupt cell walls, substantially reducing extraction time and energy consumption while preserving high-molecular-weight pectins [27,28].
- Subcritical Water Extraction (SWE), pressurized hot water acts as a tunable solvent, allowing acid-free extraction with high yields and reduced hazardous effluents [29].
- Enzyme-Assisted Extraction (EAE), application of pectinases and complementary hydrolases under mild conditions selectively solubilizes protopectin, lowering solvent requirements and preserving native structure [30].
- Pulsed Electric Field (PEF), Ohmic Heating and High-Pressure Processing (HPP), non-thermal or low-thermal methods enabling rapid release of pectins without chemical hydrolysis; these require further techno-economic validation at scale [26,31,32].

Integrating these methods within extraction–valorization chains support circular-bioeconomy objectives. Green extraction yields pectin fractions and residual solids with fewer chemical inhibitors, making the spent biomass more suitable as substrates for downstream fermentations to produce *Bacillus*-derived pectinases and other bioproducts [6,13,33]. Collectively, these approaches reduce energy use and hazardous waste, improving the overall sustainability profile of pectin valorization [26].

**Table 1:** Details of degree of esterification (DE), degree of methylesterification (DM) and yield, for pectin extracted from various sources using different methods.

Sources	Extraction Methods	DM%	DE%	Yield%	Ref.
Pomelo peels	Hot acid extraction	-	55.67	15.36	[34]
	Microwave extraction		55.34	20.43	
	Ultrasound extraction		51.42	17.21	
	Enzyme-assisted extraction		47.71	11.94	
Apple pomace	Hot acid extraction using HCl	72.02	63.80	14	[35]
	Citric acid extraction	64.05	63.42	22	
	Organic acid mixture extraction	70.25	64.55	14	
	Microwave extraction	77.0	64.80	17.6	
	Ultrasound extraction	76.75	64.18	16	
Orange Peel Waste	Extraction using HCl	-	59.37	18.73	[36]
Lemon peels	Extraction using HCl	-	82.7	13.0	[37]
Sweet lime	Hydrothermal extraction	-	71.2	23.8	[38]
Banana peel	Extraction using HCl	5.84	27.63	41.84	[39]
	Extraction using citric acid	11.52	50.27	59.57	
	Maleic acid extraction	10.25	44.88	56.45	
Watermelon Rind	Citric acid extraction	24.30	73.3	-	[40]

### 3.4. Applications of Pectins

Pectin’s gelling, thickening and stabilizing properties underpin its broad use in the food industry (e.g., jams, gels, reduced-calorie formulations) and as a source of dietary fiber [20]. In pharmaceuticals, pectins are used as biocompatible carriers in controlled-release tablets and hydrogels, modifying release kinetics and enhancing formulation stability [7,25]. Their ability to form edible films and biodegradable materials has prompted research into packaging and adhesive applications aligned with sustainability goals [41]. These application sectors create demand for targeted pectin fractions and justify efforts to optimize enzyme production. Optimized *Bacillus* fermentation platforms that produce defined enzyme blends (e.g., enriched in pectin methyl-esterase or specific lyases) therefore offer opportunities to generate tailored, high-value pectin products for food, pharmaceutical and material applications.

## 4. Overview of Pectinases

Pectinases, or pectinolytic enzymes, catalyze the depolymerization and de-esterification of the complex pectic polysaccharides that stabilize plant cell walls, unlocking a host of industrial applications from fruit-juice clarification to textile bio-scouring, wastewater remediation, and bioethanol production [42,43]. Their value derives from three convergent advantages: substrate specificity, which allows selective targeting of pectin without degrading cellulose or hemicellulose; operational versatility, with different pectinases retaining activity across a broad pH spectrum (3–11) and temperature range (20–70 °C); and environmental compatibility, replacing aggressive chemicals with biodegradable biocatalysts. Microbial pectinases, principally from fungi (e.g., *Aspergillus* spp.) and bacteria (notably *Bacillus* spp.), now account for approximately 25% of global food and industrial enzyme sales, underscoring their central role in green-processing innovations [23,24].

### 4.1. Classification of Pectinases

To align enzyme choice with application requirements, pectinases are classified along three complementary axes:

#### 4.1.1. Optimum pH

According to Bijesh and Sebastian [4], pectinolytic enzymes are broadly divided into two groups:

- Acidic pectinases (pH 3–5): Predominantly fungal enzymes, endo-polygalacturonases and pectin lyases, employed for juice extraction, wine clarification, and protoplast isolation.
- Alkaline pectinases (pH 8–11): Largely bacterial enzymes (e.g., pectate lyases from *Bacillus* spp.), used in textile degumming, pulp bio-bleaching, and alkaline wastewater treatment.

#### 4.1.2. Catalytic Mechanisms of Pectinases

**Figure 2:**
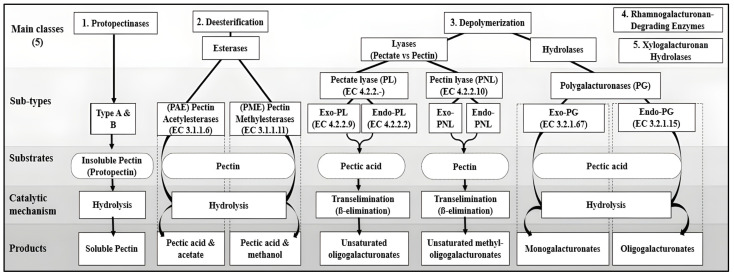
Classification of pectinases into five major classes, showing their subtypes, substrates, catalytic mechanisms, and products. The hydrolysis, trans-elimination, and depolymerization reactions are outlined with respective enzymatic groups.

Understanding the pectinase mechanism is crucial for engineering enzymes with optimal activity and stability under industrial conditions. Pectin degradation follows two complementary processes, de-esterification and depolymerization, each driven by specific enzyme subclasses (Figure 2).

In the first stage, esterases remove side-groups to prepare the homogalacturonan backbone: pectin methyl esterases (PMEs, EC 3.1.1.11) hydrolyze methyl esters to yield pectic acid and methanol, while pectin acetyl esterases (PAEs, EC 3.1.1.6) cleave acetyl esters to release acetate [14,42]. This de-esterification increases chain flexibility and exposes glycosidic bonds.

Subsequent depolymerization is carried out by two mechanistic families. Hydrolases (polygalacturonases) cleave internal α-1,4 linkages in the de-esterified regions, with endo -polygalacturonases (PGs, EC 3.2.1.15) generating oligogalacturonides and exo-PGs releasing monomeric galacturonate [44]. Pectin lyases target highly methylated pectin, producing unsaturated oligomers, whereas pectate lyases require Ca^2+^ and act on de-esterified backbones [14]. Collectively, these reactions reduce polymer length and solubilize pectin.

A tertiary suite of side-chain-specific enzymes complete the breakdown of complex pectic architectures; rhamnogalacturonan hydrolases/lyases remove RG-I and RG-II side chains, while xylogalacturonan hydrolases target xylose-substituted regions [14].

Together, these orchestrated steps ensure efficient depolymerization of pectin under the varied pH and temperature conditions encountered in biotechnological applications.

#### 4.1.3. Cellular Localization

Pectinases are also distinguished by where they reside in the cell. Extracellular pectinases are secreted directly into the medium, simplifying downstream recovery and driving their dominance in commercial enzyme markets due to low purification costs. In contrast, intracellular pectinases remain cell-bound, necessitating mechanical or chemical disruption for release, but often serve specialized metabolic functions within the organism [45].

Having established the principal classes and catalytic mechanisms of pectinases, we next examine the principal production paradigms, submerged and solid-state fermentation, which determine volumetric yield, processing footprints, and the downstream demands that shape host selection and optimization strategies.

### 4.2. Fermentation Strategies for Pectinase Production

**Figure 3:**
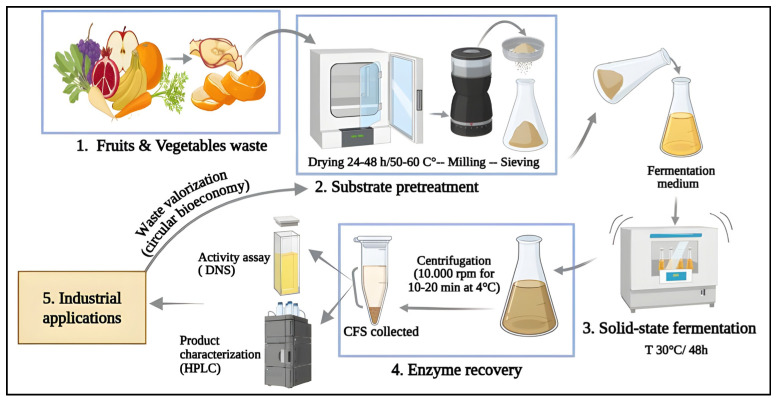
Circular solid-state fermentation process for *Bacillus* pectinases production from agro-food waste, illustrating pretreatment, fermentation, enzyme recovery, and downstream valorization.

Microbial pectinase production relies primarily on two fermentation paradigms: submerged fermentation (SmF) and solid-state fermentation (SSF) [43]. SmF, which accounts for over 90% of global enzyme output, cultivates microorganisms in dilute liquid media, enabling tight control of pH, temperature, and aeration, and facilitating automated downstream recovery. However, its reliance on high water volumes, intensive agitation, and subsequent enzyme concentration steps drives up capital and operating costs while also diluting product titers [46]. In contrast, SSF leverages low-moisture agro-industrial residues, such as citrus peels, wheat bran, sugar beet pulp, etc., to better mimic the natural habitat of filamentous fungi and certain *Bacillus* species, reducing wastewater generation and energy use (Figure 3) [47]. Moreover, reported pectinase activities under SSF often exceed those in SmF, ascribed to higher cell densities and favorable mass-transfer within the solid surfaces [48,49].

Choosing between SmF and SSF hinges on multiple interrelated factors: SmF’s scalability and process control suit fast-growing bacterial systems, particularly *Bacillus* strains secreting extracellular pectinases, whereas SSF’s resource efficiency and ability to valorize agricultural waste favor filamentous organisms. Downstream economics must also be weighed: SmF requires costly concentration of dilute broths, while SSF can impose additional costs for cell disruption if the target enzyme is intracellular. Ultimately, reactor investment, water footprint, effluent minimization, and strain physiology together determine the optimal platform [43,50,51]. Table 2 collects representative fermentation conditions and reported volumetric activities for both bacterial and fungal pectinase producers.

**Table 2:** Representative fermentation conditions and reported volumetric activities for bacterial and fungal pectinase producers.

Microorganism	Substrate	Fermentation					Pectinase Activity	Ref.
		Type	Time	T °C	pH	Agitation		
*B. amyloliquefaciens* TKU050	Wheat bran	SSF	4 days	37	6.0	100 rpm	0.76 U/mL	[48]
*B. amyloliquefaciens* SL9	Pectin	SmF	24 h	37	7.0	150 rpm	9.8 U/mL	[23]
*B. subtilis* NRRL B-4219	Hazelnut shell hydrolyzate	SmF	72 h	30	7.0	130 rpm	5.60 U/mL	[52]
*B. pumilus* NRRL B-212	Pectin	SmF	64 h	30	8.0	150 rpm	16.17 U/mL	[49]
	Sugar beet pulp	SSF	48 h	30	8.0	150 rpm	147.75 U/mL	
*B. mojavensis* I4	Carrot peels	SSF	32 h	37.5	8.0	150 rpm	64.8 U/mL	[53]
*B. tequilensis* CAS-MEI-2-33	Pectin	SmF	40 h	40	10.0	180 rpm	1370 U/mL	[54]
*B. tropicus* MCCC1A01406	Pectin	SmF	72 h	37	9.0	-	43 U/mL	[55]
*B. amyloliquefaciens* ADI2	Banana peel	SSF	48 h	28	8.38	94 rpm	2043.86 U/mL	[46]
*B. subtilis* strain Btk 27	Apple pectin	SmF	48 h	37	6.5	120 rpm	66.3 U/mL	[43]
*B. subtilis* MF447840.1	Pectin	SmF	4 days	37	7.4	120 rpm	345 ± 12.3 (U/mL)	[56]
*B. subtilis* PSE-8	Cassava peel	SSF	3 days	45	9	100 rpm	117.5 (U/mL)	[57]
*B. cereus*	Pectin	SmF	24 h	35	10.5	150 rpm	3.37 (U/mL)	[58]
*B. licheniformis*	Orange peel	SSF	120 h	37	9.5	-	219 (U/mL)	[59]
*B. subtilis* ZGL14	Pectin	SmF	72 h	40	8.0	200 rpm	734.11 (U/mL)	[60]
*Bacillus* sp. Y1	Wheat bran	SmF	72 h	37	8.2	100 rpm	40 (U/mL)	[61]
*B. safensis* M35	Citrus peel & Wheat bran	SSF	72 h	37	5.8	160 rpm	411.58 (U/mL)	[62]
*B. altitudinis* J208	Citrus peel & Wheat bran	SSF	72 h	37	6.2	160 rpm	728.74 (U/mL)	[62]
*Aspergillus niger*	Apple pomace	SSF	96 h	25	4.0	-	6.75 U/mL	[63]
*Aspergillus aculeatus* NEJC	Mango peel	SSF	8 days	40	5.5	-	1360 U/mL	[64]
*Aspergillus niger* AUMC16245	Pectin	SmF	7 days	40	7.0	200 rpm	3787.04 U/mL	[65]
*Aspergillus brasiliensis* AUMC16244	Pectin	SmF	5 days	45	7.0	200 rpm	3878.35 U/mL	[65]
*Aspergillus niveus* AUMC1624	Pectin	SmF	7 days	45	7.0	200 rpm	3572.95 U/mL	[65]
*Aspergillus foetidus*	Mango peel	SSF	96 h	30	5.5	-	228 U/mL	[66]
*Aspergillus* spp. Gm	0.5% Pectin	SmF	48 h	30	5.8	150 rpm	112 U/mL	[44]
*Saccharomyces cerevisiae*	Corn and orange peels	SSF	6 days	30	4.0	-	29.57 U/mL	[67]
*Streptomyces halstedii*	Citrus pectin	SmF	24 h	28	8.0	200 rpm	1.052 U/mL	[68]

*B.: Bacillus*, Enzyme activity (U/mL): Volumetric activity, units per milliliter of fermentation broth.

## 5. *Bacillus* vs. Fungal Pectinases

While both bacterial and fungal sources contribute substantially to commercial pectinase production, *Bacillus* species have gained increasing recognition as production hosts owing to their favorable combination of enzymatic properties, efficient fermentation characteristics, established safety profiles, and advantageous economics. The following discussion systematically contrasts fungal and *Bacillus* pectinases, highlighting the principal factors that influence host selection for industrial applications, with representative numerical examples drawn from Table 2.

### 5.1. Enzymatic Properties, Stability and Robustness

Filamentous fungi (notably *Aspergillus*) typically produce pectinases with acidic optima (pH 3.5–5.5) and moderate temperature preferences (25–45 °C), a biochemical profile that underpins their long-standing use in food-related applications [69]. Under such conditions, fungal enzymes can achieve high volumetric activities, as reported for A. brasiliensis AUMC16244 and A. aculeatus NEJC, which demonstrate activities in the acidic-to-neutral range (Table 2) [64,65]. However, fungal pectinases generally exhibit limited stability beyond these ranges, showing reduced activity under neutral to alkaline conditions or at elevated temperatures [69]. In contrast, *Bacillus*-derived pectinases display broader pH optima, often extending into the alkaline range, and enhanced thermostability (commonly 50–70 °C, with some enzymes active at higher temperatures) [4,70]. Their resilience to industrial stressors, including salts, solvents, and mechanical shear, broadens their applicability to demanding process environments such as textile bioscouring and detergent formulations, while also reducing the need for stringent process control [70].

### 5.2. Production Cycle, Substrate Flexibility, and Process Economics

Fungal fermentations, particularly SSF, often require extended cultivation periods (several days to over a week), morphology control, and nutrient-rich media, factors that increase operating costs and complicate scale-up [71]. By contrast, *Bacillus* spp. generally exhibit faster growth, efficient enzyme secretion, and adaptability to both SmF and SSF systems when using low-cost agro-industrial residues (pomace, beet pulp, banana peel). These characteristics enable shorter production cycles (commonly 24–72 h under optimized conditions), reduce downstream processing burdens, and translate into lower operational costs, making *Bacillus* particularly well-suited to decentralized and resource-efficient bioprocessing [72,73,74].

### 5.3. Genetic Engineering and Downstream Processing

Filamentous fungi often present more complex genetics and post-translational modification patterns (e.g., glycosylation), which can complicate recombinant expression and purification [75]. By contrast, *Bacillus* hosts are highly amenable to genetic manipulation, supported by well-established molecular toolkits and CRISPR-based approaches, and exhibit secretion profiles with fewer complex PTMs. These features simplify downstream purification and streamline strain engineering for tailored enzyme production [61,76,77].

### 5.4. Regulatory and Safety Considerations

Several *Bacillus* strains of industrial relevance (e.g., *B. subtilis*, *B. licheniformis*) possess recognized GRAS (or equivalent) status, which expedites regulatory approval for food and feed applications while reducing compliance costs [74]. Although many fungal strains used in pectinase production are also considered safe, certain taxa require additional safety screening (e.g., mycotoxin profiling and allergenicity assessment), which can increase development timelines and analytical burdens [78].

Taken together, these comparative features explain the increasing recognition of *Bacillus* spp. as versatile and reliable microbial platforms in industrial biotechnology. Fungal pectinases remain advantageous for acidic, food-grade processes where high raw activity on esterified pectins is critical, whereas *Bacillus*-based systems are frequently preferred for alkaline, high-temperature, or cost-sensitive industrial applications due to their secretion efficiency, short production cycles, and robustness. The following section examines *Bacillus* physiology and production strategies in light of these comparative strengths.

## 6. *Bacillus* spp. In Industrial Pectinase Production

### 6.1. Overview and Historical Milestones

The genus *Bacillus*, now encompassing over 435 species and 12 subspecies, has long stood at the forefront of industrial enzyme production. Its non-fastidious nutritional requirements, ease of large-scale cultivation, and capacity for high-level secretion of extracellular proteins laid the foundation for its adoption in the early 20th century as the premier host for protease and amylase manufacture [4,79]. Notably, *Bacillus subtilis* earned GRAS status from the Food and Drug Administration (FDA), cementing its use in food and pharmaceutical processes [80]. The advent of recombinant DNA methodologies in the late 20th century enabled overexpression of key hydrolases in *B. subtilis*, while the introduction of CRISPR-Cas9 genome editing in the 2020s has permitted precise tuning of pectinase gene clusters to enhance yield, stability, and substrate specificity [8,81].

### 6.2. Physiological and Molecular Advantages

*Bacillus* spp. pectinases distinguish themselves by combining exceptional thermostability, maintaining full catalytic activity at temperatures ≥ 60 °C, with a remarkably broad pH tolerance, from pH 4 to 10. These attributes, together with rapid bacterial growth (generation times as short as 20–30 min) and robust extracellular secretion systems, minimize downstream processing costs and accelerate production timelines. Furthermore, the well-characterized genetics of *Bacillus* facilitate advanced protein engineering: directed evolution and rational design have produced variants with enhanced resistance to metal chelators and detergent additives, overcoming two major industrial inhibition mechanisms [12,47].

### 6.3. Diversity of Pectinase-Bacillus Strains Producers

Within the *Bacillus* clade, extremophilic isolates have yielded some of the most potent pectinolytic enzymes [82] (see Table 3). For example, *Virgibacillus salarius* strain 434, sourced from hypersaline habitats, exhibits a specific activity of 104.3 U/mg at pH 9 and 70 g/L NaCl, underscoring its value in alkaline, high-salt processes [83]. Soil-derived strains such as *B. marisflavi* PSD2 and environmental isolate *Bacillus* sp. strain NRBANKI-4 further exemplify the genus’s enzymatic breadth, producing 0.61 U/mL and 6.73 U/mL of pectinase, respectively, when cultured on low-cost substrates [84,85]. Well-characterized strains such as *B. subtilis* BK-3 remain relevant, as their 33 kDa endo-polygalacturonase remains active from pH 4–10 and 30–60 °C [86], while *B. amyloliquefaciens* subsp. *plantarum* strains reveal substantial inter-strain variability in pectate-lyase activity, highlighting opportunities for strain selection and engineering [87].

### 6.4. Ecological Sources and Isolation

The widespread distribution of *Bacillus* mirrors its enzyme portfolio. Forest and agricultural soils routinely yield > 90% pectinase-positive isolates, as demonstrated by screening of 65 forest-soil strains, with 62 (95.38%) exhibiting varying degrees of pectinase activity [88]. Decayed fruit wastes, particularly orange and apple pomace, serve as rich reservoirs for pectinolytic *Bacillus*, offering sustainable feedstocks for both SmF and SSF in juice clarification applications [55]. Extremophilic niches, from saline soils to alkaline hot springs, furnish isolates whose pectinases retain activity under industrially taxing conditions of high salt, temperature, or pH, adding to the genus’s industrial versatility [82,89].

With the remarkable diversity of *Bacillus* pectinases established, the following section will delve into statistical optimization techniques, such as RSM and ANNs, employed to fine-tune fermentation parameters and maximize enzyme productivity.

**Table 3:** Biochemical and Catalytic Characteristics of Pectinases from *Bacillus* spp.

Producer Strain	Mol. Wight	Enzyme Type	Opt. pH	Opt. T °C	Specific Enzyme Activity	Stability (pH/T°C)	Kinetics		Applications	Ref.
							Km	Vmax		
*Virgibacillus salarius* Strain 434	68 kDa	Pectinase	9	40	104.3 U/mg	7.0–9.5 -	0.38 mg/mL	120 U/mg	Pretreatment of wastewater from textile and paper industries	[83]
*Bacillus halodurans M29*	39 kDa	Pectinase	10	80	142 U/mg	9.5–10.5	4.1 mg/mL	351 U/mg	-	[70]
*Bacillus* sp. DT7	106 kDa	Pectin lyase	8.0	60	1433 U/mg	7.5–8.5 40–60 °C	-	-	In textile industry, plant tissue maceration and wastewater treatments	[90]
*B. subtilis* strain BK-3	33 kDa	Pectinase	5	50	143.77 U/mg	4–10 30–60 °C	0.4770 mg/mL	43.46 U/mL	Clarification of fruit juice	[86]
*Bacillus* sp. strain B58-2	-	Pectate lyase	8.5	50	2433.26 U/mg	-	-	-	Ramie degumming	[91]
*B. pumilus*	60 kDa	Pectinase	8.0	60	156.5 U/mg	-	-	-	Clarification of fruit juice	[92]
*B. tropicus* P-3	-	Alkaline pectinase	9.0	37	65 U/mg	-	2.2 mg/mL	44 U/mg	Pretreatment of the fabrics	[55]
*B. licheniformis* KIBGE IB-3	-	Polygalacturonase	7	37	1118.12 U/mg	5–9 -	-	-	-	[93]
*Bacillus* sp. strain BR1390	104 kDa	Polymethylgalacturonase	6	60	222.6 U/mg	5–8 -	2.51 mg/mL	0.066 µmol/ min	Applications in the fruit juice industry	[94]
*B. subtilis* SS	-	Pectinase	9.5	70	5.943 U/g	7–10 55–70 °C	-	-	Pulp and Paper Industry	[95]
*B. subtilis* 15A-B92	14.4 kDa	-	4.5	50	99.6 U/mg	-	1.72 mg/mL	1609 U/g	Clarification of orange and apple juices	[96]
*B. subtilis* ZGL14	65 kDa	-	8.6	50	52,372.52 U/mg	-	-	-	-	[60]
*Bacillus* sp. ZJ1407	23 kDa	-	5.0	37	110.47 U/mg	3–5 80–90 °C	-	-	-	[60]
*B. subtilis* PB1	43.1 kDa	Pectate lyase	9.5	50	1252.82 U/mg	5–11 -	0.312 mg/mL	1248 U/mL	Flue-cured tobacco leaves	[97]
*B. pumilus* DKS1	35 kDa	Pectate lyase	8.5	75	6200 U/mg	-	0.44 mg/mL	909 U	Fibre degumming	[98]
*B. clausii*	-	Pectate lyases	10.5	70	936.2 U/mg	-	0.54 mg/mL	-	Ramie degumming	[99]

Specific activity (U/mg): units per milligram of total protein, U/g: Units per gram of solid substrate in SSF.

## 7. Statistical Optimization of *Bacillus* spp. Pectinase Production

### 7.1. RSM, ANNs and DoE for Pectinase Yield Optimization

Statistical optimization has become a crucial tool in enhancing the production of pectinases in *Bacillus* spp., a process that is highly influenced by several environmental and nutritional factors [59,100]. Various advanced techniques, including Response Surface Methodology (RSM), Artificial Neural Networks (ANN), and Design of Experiments (DoE), have demonstrated their effectiveness in identifying the optimal conditions that maximize enzyme yield [101]. These methods enable researchers to systematically evaluate and optimize multiple variables simultaneously, rather than adjusting them one at a time, which significantly improves efficiency and accuracy in determining ideal fermentation conditions [15].

The RSM is a statistical technique that utilizes a set of mathematical and statistical tools to model and analyze the relationships between multiple input factors and the response variable, in this case, pectinase yield [102]. The core advantage of RSM lies in its ability to determine the optimal levels of factors such as substrate concentration, temperature, pH, and inoculum size through a series of designed experiments. RSM generates a response surface that visually represents how changes in each factor influence enzyme production, allowing researchers to pinpoint the ideal combination of conditions. In many studies, RSM has been employed to investigate the interactions between key parameters and their combined effects on pectinase activity, enabling the identification of the most favorable production environment. For instance, it has been used to optimize pectinase production from *Bacillus subtilis*, where pH, temperature, and carbon source concentration were optimized to increase enzyme yield [59].

ANNs are computational models inspired by the way biological neural networks process information. In the context of pectinase production, ANNs offer a powerful approach to predict enzyme yield based on complex and nonlinear relationships between the process parameters [103]. Unlike traditional linear models, ANNs can model intricate interactions between factors that might not be immediately apparent, making them particularly useful in fermentation processes where numerous variables interact in complex ways. By training neural networks with experimental data, researchers can predict enzyme production under various conditions without the need for exhaustive experimental setups. ANNs have been successfully applied in optimizing pectinase production in *Bacillus* sp., where they help predict how changes in temperature, pH, and other factors impact enzyme yield. This predictive capability allows for more efficient experimentation, potentially reducing time and costs.

The DoE is a statistical approach used to plan and conduct experiments efficiently. It involves systematically varying experimental factors to observe their effects on the response variable, ensuring that all possible interactions between factors are considered. Unlike traditional trial-and-error methods, DoE helps to identify not only the main effects of individual factors but also their interactions, leading to a deeper understanding of the fermentation process. For example, by using fractional factorial designs, researchers can test several parameters simultaneously while minimizing the number of experiments needed. This approach is particularly useful when optimizing processes such as pectinase production, where multiple factors must be balanced. DoE has been used to optimize conditions such as inoculum size, aeration rate, and nutrient levels for *Bacillus* sp., resulting in significant improvements in pectinase productivity [104].

Together, these optimization techniques allow for a more nuanced approach to enhancing pectinase production. By carefully controlling and fine-tuning factors like substrate concentration, pH, temperature, and inoculum size, researchers can maximize the efficiency of *Bacillus* spp. fermentation systems. These methods not only improve enzyme yield but also enable the development of more cost-effective and sustainable production processes, making them indispensable tools for the industrial-scale production of pectinases.

For instance, RSM has been used in numerous studies to design experiments and model the interactions between different production parameters. ANNs have also gained popularity in recent years, particularly for their ability to predict complex non-linear relationships in fermentation processes. These techniques allow for the identification of optimal conditions that maximize pectinase activity and productivity while minimizing resource usage. Researchers have observed that adjusting parameters like temperature and pH within optimal ranges can result in significant improvements in enzyme yield. Moreover, combining these methods with metabolic engineering approaches can lead to the further enhancement of *Bacillus* spp. pectinase production.

### 7.2. Strain-Specific Responses and Process Determinants of Pectinase Yield

Pectinase yield from *Bacillus* spp. is governed by an interaction between strain-intrinsic properties (genetics, secretion capacity, regulatory networks) and operational variables (pH, temperature, substrate composition, inoculum, agitation, aeration, medium composition) (See Table 2 for representative strains and activities) [105]. Understanding these interactions is essential for designing reproducible, scalable fermentations and for aligning statistical optimization tools (RSM, ANN, DoE) with strain physiology [23,106].

#### 7.2.1. pH and Temperature

Pectinase expression and activity are highly pH- and temperature-dependent and vary among strains. Many *Bacillus* strains show maximal production in the slightly acidic range (pH 5.0–6) and moderate temperatures (30–40 °C), which balances cellular growth and enzyme stability [58,73,107]. However, thermotolerant species such as *B. licheniformis* can maintain high expression at elevated temperatures (up to 50 °C) and toward neutral-alkaline pH, which is advantageous for specific industrial applications [59,108]. These differences require strain-specific optimization rather than a one-size-fits-all approach.

#### 7.2.2. Inoculum Size and Seed-Culture State

Inoculum density and physiological state critically influence fermentation kinetics and enzyme yield [109]. Typical inoculum ranges reported for *Bacillus* fermentations are ~1–5% (*v*/*v*), with strain-dependent optima; too small inoculum prolongs the lag phase, while excessive inoculum can cause rapid nutrient depletion, oxygen limitation and lower per-cell productivity [110]. For example, *B. subtilis* has been shown to produce more pectinase when the inoculum size is maintained at around 2%, as it ensures adequate growth without overwhelming the fermentation medium [86]. The physiological state (growth phase) of the seed culture also affects timing and magnitude of enzyme secretion and should therefore be standardized during optimization [100].

#### 7.2.3. Agitation and Aeration

Agitation and aeration determine oxygen transfer, mixing and mass-transfer rates, which directly affect aerobic *Bacillus* metabolism and pectinase production [111]. Optimal agitation ensures homogeneity without imposing damaging shear; excessive agitation may induce foaming and cell stress, while insufficient agitation produces oxygen gradients that limit productivity [112,113]. Reported optimal agitation rates are strain- and reactor-dependent (e.g., 150 to 200 rpm in stirred-flask studies), and agitation must be co-optimized with aeration rates during scale-up [114].

#### 7.2.4. Substrate Composition (Carbon Source Effects and Induction)

Substrate composition exerts a strong, strain-dependent effect on pectinase expression. Readily metabolizable monosaccharides (glucose, fructose) commonly trigger CcpA-mediated carbon catabolite repression (CCR) in *Bacillus*, down-regulating pectinolytic genes; by contrast, pectin-rich and structurally complex carbohydrates act as inducers of pectinase biosynthesis (e.g., pectin induction of the *rhiL* promoter in *B. subtilis*) [115,116]. Empirical studies illustrate this: *B. pumilus* NRRL B-212 attains higher titres on sugar-beet pulp (rich in pectin and galacturonate) [49], whereas *B. subtilis* benefits from mixed-substrate regimes in which simple sugars support biomass accumulation while pectinaceous residues induce enzyme synthesis [43]. Agro-residues (citrus peel, apple pomace, beet pulp) therefore often outperform refined sugars by providing both inducers and auxiliary nutrients, reducing fermentation cost and aligning with circular-bioeconomy objectives [117] (see Table 2).

#### 7.2.5. Medium Composition and Non-Carbon Factors

Beyond carbon, nitrogen source (organic vs. inorganic), mineral salts and metal cofactors (Ca^2+^, Mg^2+^, Mn^2+^, Fe^2+^), trace vitamins, and dissolved oxygen interact with carbon metabolism to shape enzyme profiles and titres [47,70]. Synthetic industrial media (e.g., glucose, yeast extract and peptone) give predictable growth but may require supplementation to avoid CCR and achieve target titres; by-product substrates can reduce supplementation needs but introduce feedstock heterogeneity that must be managed during scale-up [43,118].

#### 7.2.6. Strain Robustness, Scale-Up and the Role of Statistical Optimization

Strain robustness to environmental and process stressors (osmotic, oxidative, shear) is crucial for large-scale implementation [110,119]. Robust strains such as certain *B. subtilis* isolates maintain productivity under fluctuating conditions, whereas less tolerant strains may require tighter process control or strain improvement [120,121]. Statistical optimization methods (RSM, ANN, DoE) remain powerful to identify optimum process windows, but their outputs are only meaningful when aligned with strain physiology and validated at scale [121]. Collectively, strain selection and integrated process optimization determine whether laboratory gains translate into industrial metrics of productivity and cost-effectiveness [120].

## 8. Industrial Significance of *Bacillus* Pectinases

Pectinases from *Bacillus* species offer an eco-friendly alternative to harsh chemical treatments, reducing environmental pollution and process costs (Table 4) [122].

### 8.1. pH-Dependent Industrial Applications of Pectinases

**Figure 4:**
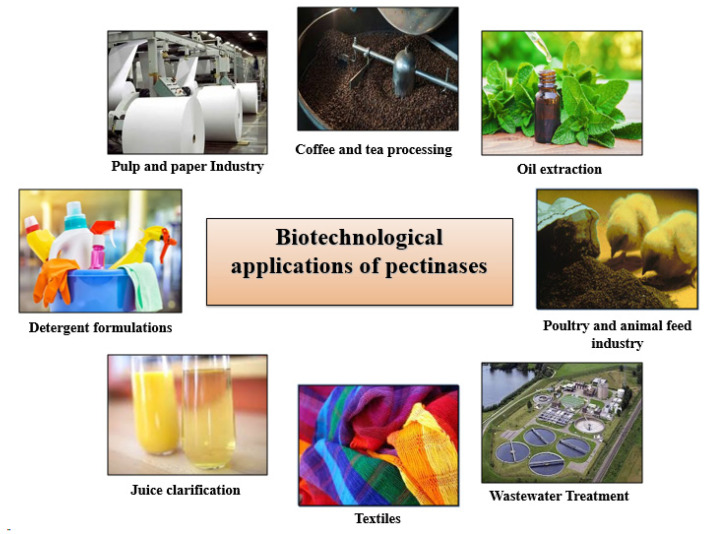
Industrial applications of *Bacillus* pectinases.

Pectinases are categorized as alkaline or acidic based on their optimum pH, with each type processing distinct commercial applications (Figure 4).

#### 8.1.1. Alkaline Pectinases

*Bacillus* spp. are leading producers of alkalophilic pectinases, enzymes ideally suited for high-pH industrial processes [125].

- Textile Bioscouring and Degumming

Bioscouring: Alkaline pectinases offer an eco-friendly alternative to traditional chemical scouring. This enzymatic process minimizes energy consumption and generation of toxic effluent by selectively removing pectin-based impurities from cotton fibers [126]. For example, pectinases from *B. paralicheniformis* significantly enhance cotton hydrophilicity and dye uptake without damaging the fiber integrity [127].

Degumming: The efficient removal of non-cellulosic gums from bast fibers like ramie is another critical application. Alkaline pectinases from strains such as *Bacillus* sp. B58-2 and *B. clausii* effectively degrade pectic substances under alkaline conditions and elevated temperature, yielding smoother, higher-quality fibers [91,99].

Implementing these enzymatic processes at scale relies on two key strategies:

Enzyme Immobilization: Immobilization of producer cells, such as *B. pumilus*, on solid supports enhances enzyme yield and operational stability, improving process economics and enabling continuous large-scale applications [128].

Process Optimization: Maximizing the effectiveness of pectinase treatments requires precise optimization of critical parameters, including pH, temperature, enzyme concentration, and treatment time [60,129].

- Pulp and Paper Biobleaching

*Bacillus*-derived pectinases, particularly in combination with xylanases and other lignocellulolytic enzymes, are increasingly adopted in the pulp and paper industry for efficient fiber processing and eco-friendly bleaching. For instance, a xylano-pectinolytic enzyme cocktail from *B. amyloliquefaciens* ADI2 improved pulp brightness by 11.25% when applied to oil palm empty fruit bunches, significantly reducing the demand for chemical bleaching agents and associated organochlorine pollutants [130]. Similarly, enzymes from *Bacillus subtilis* and *Bacillus pumilus*, noted for their thermostability and alkalophilicity, decreased the permanganate number by 84% and the kappa number by 16% during kraft pulp treatment, enhancing brightness while minimizing chlorine dioxide consumption [131]. The efficacy of bleaching is further augmented by enzyme cocktails that incorporate pectinases with laccases and mannanases. Notably, a formulation from *B. tequilensis* LXM 55 achieved a brightness increase of 11.59% and a kappa number reduction of 49.35%, enabling chlorine reductions of up to 40% [132]. Beyond reducing adsorbable organic halogens (AOX), these enzyme systems also demonstrate significant reductions in chemical oxygen demand (COD), as evidenced by studies with pectinase from *Bacillus subtilis* SS [95]. Beyond bleaching, *Bacillus* pectinases facilitate effective fiber digestion by hydrolyzing pectic substances in plant cell walls. This application is particularly valuable for processing non-woody materials such as *mitsumata* bast; enzymatic maceration by alkalophilic *Bacillus* strains yields pulp with improved tensile strength and burst index [95,133]. Synergistic interactions between pectinases and other hemicellulases, such as xylanases, enhance pulp permeability, lignin removal, and brightness uniformity [131,132].

- Detergent Formulations

Recent advances position *Bacillus* spp. at the forefront of bio-based detergent development, capitalizing on their dual capacity to secrete robust enzymes and potent biosurfactants [134]. Specifically, the co-production of pectinases and rhamnolipid-like surfactants by *Bacillus subtilis* BKDS1, demonstrated at 1 L fermentation scale using pineapple stem hydrolysate, highlights the potential of this genus for scalable and cost-effective detergent biotechnology [121]. Protein engineering has yielded “extremozymes” that retain over 80% activity at pH 10–11 and 60 °C while exhibiting resistance to common detergent chelators. For example, chelate-resistant type I pullulanase shows exceptional stability against detergent inhibitors, broadening enzyme compatibility with surfactants, builders, and bleaching agents [135]. Furthermore, integrating pectinases with complementary *Bacillus* proteases improves the removal of mixed organic stains without compromising fabric integrity. The biodegradable nature of these enzymes and their co-produced biosurfactants markedly reduces environmental impact compared to conventional chemical formulations [136,137]. Emerging patents on alkaline, thermostable pectinases and integrated enzyme-biosurfactant platforms underscore a growing industrial commitment to sustainable, high-performance detergent technologies [134].

- Coffee and Tea Processing

*Bacillus*-derived pectinases serve as powerful biocatalysts for enhancing coffee and tea quality through controlled and efficient fermentation. In coffee production, pectinolytic enzymes from strains such as *B. subtilis* and *B. cereus* accelerate mucilage removal from beans, reducing processing time from days to hours (e.g., 6.4 h for ripe beans at 0.3 mL/L enzyme) while ensuring more consistent bean quality [138]. This targeted hydrolysis also modulates the developing flavor and aroma profile by liberating reducing sugars and phenolics, resulting in higher caffeine levels and a richer volatile spectrum in the final roast [139]. In tea processing, *Bacillus* pectinases facilitate cell-wall loosening, which enhances leaf oxidation, color extraction, and foam control in instant formulations [140]. Applying pectinases isolated from agro-industrial residues (e.g., decayed orange peel) increases flavonoid and total phenol content, thereby improving the antioxidant capacity and extending the shelf life of tea products [141]. Moreover, enzyme preparations produced via SSF on low-cost substrates (e.g., cocoa pod husk) lower production costs by valorizing waste materials, while maintaining tea grade and sensory attributes [142]. Compared to harsh chemical treatments, these enzymatic approaches are not only more effective but also align with sustainable processing goals by reducing environmental impact [143].

- Oil Extraction

*Bacillus*-derived pectinases enhance oil recovery from plant matrices by hydrolyzing pectic emulsifiers that entrap essential oils. For example, *B. mojavensis* I4 produced 64.8 U/mL of pectinase on carrot peel under optimized conditions (6.5% substrate, 0.3% NH_4_Cl, 3% inoculum, 32 h), leading to increased oil yields [53]. Similarly, pectinases isolated from orange and grapefruit peels, exhibiting activities up to 3.17 U, boosted sesame oil recovery by 3% compared to non-enzymatic methods [144]. This enzymatic pretreatment parallels juice-extraction processes, facilitating phase separation and reducing solvent use [145]. Crucially, producing pectinases on low-cost agro-residues (e.g., banana peels) further lowers production costs and aligns with green bioprocessing principles [146]. Although strain-specific variability necessitates tailored optimization, these studies underscore the potential of *Bacillus* pectinases to demonstrate higher yields, purer oils, and more sustainable extraction workflows.

- Poultry and Animal Feed Industry

Incorporating *Bacillus* pectinases into poultry and livestock diets enhances nutrient bioavailability by hydrolyzing pectic polysaccharides that increase digesta viscosity and hinder absorption [147]. Supplementation with these enzymes improves the digestibility of alternative feedstuffs such as distillers’ dried grains with solubles (DDGS) and promotes a balanced gut microbiota by suppressing pathogens like *Clostridium perfringens* [147]. Studies in poultry have documented improved intestinal integrity and overall health, leading to higher weight gain and feed conversion efficiency [148].

- Purification of Plant Viruses

Alkaline pectinases from *Bacillus* spp. are invaluable for isolating phloem-limited plant viruses, which are otherwise challenging to recover due to their embedding within vascular cell walls. By selectively hydrolyzing pectin networks, these enzymes liberate virions with minimal damage, yielding higher titers for downstream assays [149]. For example, pectino-cellulolytic preparations increased rice tungro virus complex (RTV-C) yields by 1.39 to 3.12-fold compared to traditional extraction methods [149]. Likewise, “Driselase” a *Bacillus* derived enzyme cocktail, boosted Potato Leafroll Virus (PLRV) recovery from 0.5 mg/kg to 4.7 mg/kg and Tobacco Necrotic Dwarf Virus (TNDV) from 0.5 mg/kg to 1.3 mg/kg without compromising infectivity [150]. Following enzymatic maceration, standard clarification steps (e.g., centrifugation and polyethylene glycol precipitation) can be applied to concentrate and purify viral particles. This combined approach not only preserves viral integrity but also provides sufficient material for high-resolution molecular and structural analyses, underscoring the critical role of *Bacillus* pectinases in advancing plant virology research. Notably, alkaline pectinases from *Bacillus* sp. DT7 have also demonstrated exceptional efficacy in virus purification applications [143].

#### 8.1.2. Acidic Pectinases and Their Industrial Applications

Acidic pectinases, which exhibit optimal activity at pH 3–6, play indispensable roles across food, textile, paper, and waste-treatment industries [5]. Their ability to hydrolyze high-methoxyl pectins under mild acidic conditions improves process yields and product quality while enabling the valorization of agro-industrial residues for enzyme production, supporting circular economy initiatives.

- Juice Clarification

In fruit juice manufacturing, acidic pectinases drastically reduce viscosity and turbidity by depolymerizing methyl-esterified pectin, thereby enhancing juice yield and clarity. For instance, Pavlović, et al. [151] demonstrated that an endo-pectin lyase (BvPelB) from B. velezensis 16B, applied at 37 °C and pH 5.5, improved the clarity of fruit juices. Chaudhari, et al. [152] further showed that producing acidic pectinase on low-cost substrates like sweet-lemon peel and lemon yields high activities, underscoring the feasibility of agro-waste valorization. Patrício, et al. [153] quantified these benefits, reporting turbidity reductions > 98%, viscosity reductions > 80%, and juice yield increases of up to 280% in grape juice trials, confirming acidic pectinases as high-impact clarifying agents.

- Wine Stabilization

Cloudiness and sedimentation in wines arise from high-molecular-weight pectins; endo-polygalacturonases from *Bacillus* spp. resolve these issues by cleaving pectin polymers at pH 3–4. Immobilized preparations, such as those via noncovalent adsorption onto polyamide microparticles, retain >80% activity over multiple batches and accelerate fining times by up to 50% [154,155]. Beyond clarity, pectinase treatment liberates bound phenolics and volatile aromatics, subtly enhancing mouthfeel and aroma profiles, though careful dosing is critical to avoid off-flavor development [156].

- Food Processing: Fruit Peeling, Canning, and Product Stabilization

Acidic pectinases from *Bacillus* spp. have advanced fruit peeling and canning technologies by selectively hydrolyzing cell wall pectins under mildly acidic conditions, enabling rapid and uniform skin removal without compromising fruit integrity. Enzymes produced by *B. subtilis* and *B. licheniformis* exhibit high catalytic activity across pH 4–6 and temperatures of 30–60 °C, resulting in more efficient peeling and improved surface quality in tomatoes, apples, and citrus [86,157]. Additionally, *B. velezensis* pectinases have demonstrated enhanced cell wall degradation, leading to cleaner and more consistent peeling outcomes [151]. Beyond peeling and canning, *Bacillus* pectinases are employed in product stabilization, particularly in juices and jams, by preventing pectin-induced gelation and sedimentation. Notably, a pectinase from *B. subtilis* 15A-B92 completely hydrolyzed citrus pectin at 37 °C, resulting in clarified, haze-free juice [96].

### 8.2. Bacillus Pectinases in Bioremediation and Environmental Sustainability

The non-toxic, biodegradable nature of *Bacillus*-derived pectinases makes them attractive eco-friendly alternatives to conventional chemical treatments for managing pectin-rich industrial effluents and contaminated soils [7]. Unlike physicochemical remediation methods, which often generate secondary pollutants and impose high energy and chemical costs, pectinolytic *Bacillus* spp. or their purified enzymes efficiently depolymerize complex pectin matrices, reducing effluent viscosity and organic load without toxic byproducts [158]. These enzymes operate optimally between 30–60 °C and pH 6–9, conditions that are commonly encountered in agro-food, textile, and pulp-and-paper waste streams [60,159,160]. When applied synergistically with cellulases and xylanases, *Bacillus* pectinases have been shown to enhance biomass saccharification by up to 1.9-fold, significantly improving biodegradation kinetics [160]. Furthermore, their application in the treatment of high-strength wastewater from food waste recycling has demonstrated high removal efficiencies of macromolecular pollutants such as proteins, lipids, and polysaccharides [161]. Beyond wastewater treatment, these enzymes also contribute to soil remediation by accelerating the degradation of pectin-rich agricultural residues, thereby improving nutrient cycling, reducing phytotoxicity, and enhancing overall soil health [162]. The use of low-cost agro-industrial by-products as substrates for enzyme production adds an additional dimension of sustainability, facilitating both waste valorization and cost-effective biocatalyst synthesis [7]. Collectively, these attributes underscore the significant potential of *Bacillus*-derived pectinases to advance green technologies and support circular bioeconomy frameworks through cleaner, safer, and more sustainable waste management solutions.

## 9. Challenges & Future Directions

While *Bacillus*-derived pectinases have already demonstrated remarkable versatility, from green extraction synergies to textile bio-scouring and bio-bleaching, several critical challenges must be addressed to unlock their full industrial potential.

### 9.1. Integration of Upstream and Downstream Processes

True circularity will require co-locating green pectin extraction (e.g., UAE, SWE, EAE) with on-site *Bacillus* fermentation, enabling direct hydrolysis of cell walls and in situ enzyme recovery. Such “zero-waste” biorefineries have been conceptually demonstrated [6,33] but remain to be piloted at scale.

Agro-residues vary widely in composition, moisture, and inhibitory compounds. Developing robust pre-treatment and adaptive fermentation protocols, potentially guided by real-time sensors and digital twins, will be essential to maintain consistent pectinase yields [110].

### 9.2. Economics of Low-Cost Production

Although fruit peels, sugar beet pulp, and other wastes are attractive, their availability, transport, and storage costs can erode savings. Life-cycle techno-economic analyses must compare “no-cost” substrates against centralized commodity feeds to identify true cost minima [7].

Advanced bioreactor designs, such as solid-state packed beds, membrane-aerated columns, or continuous stirred tank reactors with cell retention, can boost volumetric productivity and reduce downstream concentration costs [110].

### 9.3. Enzyme Engineering & Strain Diversification

Extremophilic strains from underexplored niches (e.g., mining effluents, saline deserts) may harbor pectinases with superior stability or multifunctionality [23]. Comprehensive genomic mining and metagenomic screening will accelerate this discovery pipeline.

Machine-learning-guided mutagenesis and CRISPR*Cas9-mediated genome editing can tailor active sites for enhanced thermostability, altered pH-optima, and resistance to detergents or heavy metals [9]. Integrating molecular dynamics simulations with high-throughput screening will shorten the R&D cycle.

### 9.4. Process Optimization and Scale-Up

Beyond traditional RSM or ANNs, digital-twin models that fuse real-time process data with mechanistic enzyme kinetics will enable self-optimizing bioprocesses. This approach can dynamically adjust temperature profiles, aeration rates, or substrate feeds to maintain peak activity [60].

Combining covalent, encapsulation, and affinity-tag strategies can improve enzyme half-life and facilitate continuous operations. Systematic studies comparing retention methods under industrial shear and solvent stresses are urgently needed [156].

### 9.5. Expanding Application Horizons

Tailored pectinases could generate novel oligosaccharides for biodegradable polymers or functional food ingredients. Co-cultivation with other biocatalysts (e.g., polygalacturonate lyases, trans-glycosylases) may yield value-added co-products in a single bioreactor.

In-field formulations of pectinases, possibly delivered via spore formulations or root-colonizing *Bacillus*, offer routes to in situ soil conditioning, plant-pathogen control, and enhanced nutrient release from crop residues [23].

## 10. Conclusions

*Bacillus*-derived pectinases represent a powerful class of biocatalysts with wide-ranging industrial relevance, offering sustainable alternatives to conventional chemical-intensive processes. Their robustness, thermostability, and adaptability to both acidic and alkaline environments make them particularly suitable for applications in the food, textile, paper, and environmental sectors. This review has consolidated the state-of-the-art in *Bacillus* pectinase research, spanning from green extraction of their primary substrate to advanced enzyme production and application strategies. Innovations in enzyme fermentation optimization (SmF, SSF, RSM, ANN) and the valorization of agro-industrial wastes have collectively advanced the techno-economic feasibility of *Bacillus*-based pectinase bioprocesses. Despite substantial progress, key challenges persist, namely in maximizing enzyme yields from heterogeneous substrates, enhancing catalytic stability under industrial conditions, and reducing production costs. To transition *Bacillus* pectinases from laboratory to truly sustainable industrial mainstays, future work must weave together advanced enzyme discovery, computationally driven process design, and holistic techno-economic validation. By doing so, the next generation of pectinase biorefineries will not only replace harsh chemistries but also underpin circular-economy platforms across food, materials, and environmental sectors. Altogether, *Bacillus* pectinases are well-positioned to support the transition toward a circular bioeconomy, driving innovation in sustainable industrial biotechnology.

## Figures and Tables

**Table 4:** List of companies producing commercial pectinases (a: [122], b: [123], c: [124]).

Product Trade Name	Manufacturer	Country
Pectinex™ ^a^	Novo Nordisk (Now Novozymes)	Denmark
Pectinase Enzyme ^a^	Carolina Biological Supply Co.	United States of America
Pectinase ^a^	CCM International Ltd.	Various
Panzym ^b^	C.H. Boehringer Sohn,	Ingelheim, West Germany
Ultrazyme ^b^	Ciba-Geigy, A.G.	Basel, Switzerland
Pectolase ^b^	Grinsteelvaeket	Aarthus, Denmark
Sclase ^b^	Kikkoman Shoyu, Co.	Tokyo, Japan
Pectinex ^b^	Schweizerische Ferment, A.G.	Basel, Switzerland
Rapidase, Clarizyme ^b^	Societe Rapidase, S.A.	Seclin, France
Klerzyme ^b^	Wallerstein, Co.	Des Plaines, United States of America
Pectinol, Rohament ^b^	Rohm, GmbH	Darmstadt, West Germany
Pectinase ^c^	Biocatalysts Ltd.	Cardiff, United Kingdom
Sunson Industry Group Co. Ltd. ^c^	Sunson^®^ PEC-pectinase	Yinchuan, China
Yakult Pharmaceutical Industry Co. Ltd. ^c^	Macerozyme, Pectinase	Tokyo, Japan
Esseco Group ^c^	EnartisZym	San Martino, Italy
Megazyme International ^c^	Pectate lyase, Pectinase	Wicklow, Ireland

## Data Availability

No new data were created or analyzed in this study.

## Abbreviations

The following abbreviations are used in this manuscript:

ANN	Artificial Neural Network
*B.*	*Bacillus*
CCD	Central composite design
CCR	Carbon catabolite repression
DoE	Design of Experiments
DM	Degree of methyl-esterification
DE	Degree of esterification
EAE	Enzyme-Assisted Extraction
HG	Homogalacturonans
HPP	High-Pressure Processing
MAE	Microwave-Assisted Extraction
OFAT	One factor at a time
PEF	Pulsed Electric Field
PG	Polygalacturonase
RG	Rhamnogalacturonans
RSM	Response surface methodology
SmF	Submerged fermentation
SSF	Solid-state fermentation
SWE	Subcritical water extraction
UAE	Ultrasound-Assisted Extraction
